# Vancomycin resistant enterococcus risk factors for hospital colonization in hematological patients: a matched case-control study

**DOI:** 10.1186/s13756-023-01332-x

**Published:** 2023-11-13

**Authors:** Marianna Meschiari, Shaniko Kaleci, Martina Del Monte, Andrea Dessilani, Antonella Santoro, Francesco Scialpi, Erica Franceschini, Gabriella Orlando, Adriana Cervo, Morselli Monica, Fabio Forghieri, Claudia Venturelli, Enrico Ricchizzi, Johanna Chester, Mario Sarti, Giovanni Guaraldi, Mario Luppi, Cristina Mussini

**Affiliations:** 1 Department of Infectious Diseases, Azienda Ospedaliero-Universitaria, University of Modena and Reggio Emilia, Via del Pozzo 71, Modena, 41122 Italy; 2 Clinical and Experimental Medicine, University of Modena and Reggio Emilia, Via del Pozzo 71, Modena, 41122 Italy; 3 Clinical Microbiology Laboratory, University of Modena and Reggio Emilia, Via del Pozzo 71, Modena, 41122 Italy; 4 Agenzia Sanitaria e Sociale Regionale Emilia-Romagna, Viale Aldo Moro 21, Bologna, 40127 Italy; 5 Department of Dermatology, University of Modena and Reggio Emilia, Modena, 41121 Italy; 6 Section of Hematology, Department of Surgical and Medical Sciences, AOU Policlinico, University of Modena and Reggio Emilia, Modena, Italy

**Keywords:** Vancomycin resistant Enterococcus, Hematological, Risk factors

## Abstract

**Background:**

Vancomycin-resistant enterococcus (VRE) was the fastest growing pathogen in Europe in 2022 (+ 21%) but its clinical relevance is still unclear. We aim to identify risk factors for acquired VRE rectal colonization in hematological patients and evaluate the clinical impact of VRE colonization on subsequent infection, and 30- and 90-day overall mortality rates, compared to a matched control group.

**Methods:**

A retrospective, single center, case–control matched study (ratio 1:1) was conducted in a hematological department from January 2017 to December 2020. Case patients with nosocomial isolation of VRE from rectal swab screening (≥ 48 h) were matched to controls by age, sex, ethnicity, and hematologic disease. Univariate and multivariate logistic regression compared risk factors for colonization.

**Results:**

A total of 83 cases were matched with 83 controls. Risk factors for VRE colonization were febrile neutropenia, bone marrow transplant, central venous catheter, bedsores, reduced mobility, altered bowel habits, cachexia, previous hospitalization and antibiotic treatments before and during hospitalization. VRE bacteraemia and *Clostridioides difficile* infection (CDI) occurred more frequently among cases without any impact on 30 and 90-days overall mortality. Vancomycin administration and altered bowel habits were the only independent risk factors for VRE colonization at multivariate analysis (OR: 3.53 and 3.1; respectively).

**Conclusions:**

Antimicrobial stewardship strategies to reduce inappropriate Gram-positive coverage in hematological patients is urgently required, as independent risk factors for VRE nosocomial colonization identified in this study include any use of vancomycin and altered bowel habits. VRE colonization and infection did not influence 30- and 90-day mortality. There was a strong correlation between CDI and VRE, which deserves further investigation to target new therapeutic approaches.

**Supplementary Information:**

The online version contains supplementary material available at 10.1186/s13756-023-01332-x.

## Background

Enterococci are Gram-positive, facultatively anaerobic oval cocci which are part of the human gut microbiota [1]. The two most frequently isolated species are *Enterococcus faecium* and *Enterococcus faecalis*. They are both low virulent bacteria, but can lead to severe infections, such as bloodstream (frequently polymicrobial), intra-abdominal, urinary tract, surgical site, central nervous system infections, and endocarditis (mostly *E. faecalis*) [2]. *Enterococcus spp* has an intrinsic resistance to different classes of antibiotics, including β-lactams, often in combination with aminoglycosides [3], quinolones, tetracyclines and glycopeptides (including vancomycin) [4].

The mechanism responsible for glycopeptides resistance involves modifications in the synthesis of peptidoglycan. D-Ala residues on peptidoglycan precursor can either be replaced by a D-Lactate or by a D-Ser residue [5]. To date, 9 genes causing vancomycin resistance have been identified, including vanA, vanB, vanC, vanD, vanE, vanG, vanL, vanM, VanN. VanC gene is responsible for the production of intrinsic resistance unique to *Enterococcus gallinarum* and *Enterococcus casseliflavus* [6]. VanA and VanB are the most commonly identified genes in clinically isolated strains of *Enterococci* and are predominantly carried by *E. faecium*. Expression of VanA and VanB genes is regulated following bacterial exposure to glycopeptides [7]. Vancomycin induces both VanA and VanB expression, while teicoplanin induces VanA only [5]. These genes can transfer between enterococci by means of plasmid transfer and transposon integration. Vancomycin-resistant enterococci (VRE) have been shown to arise *de novo* in the gastrointestinal tract as the result of horizontal gene transfer from anaerobic flora to *E. faecium* [3, 4].

The recent increased incidence of VRE infections in the nosocomial setting is of great concern [8]. In 2017, WHO listed VRE as a “high priority pathogen”, estimated as responsible for around 30% of all healthcare-associated enterococcal infections [8]. The European Centre for Disease Prevention and Control (ECDC) announced that *E. faecium* was the fastest growing pathogen in Europe in 2022 (+ 21%). However, compared to other gram-negative pathogens, the clinical relevance of this prevalence is currently unclear [9].

Due to VRE’s ability to adapt and persist in the hospital environment, nosocomial spreading can cause dangerous epidemic outbreaks [10]. These outbreaks are attributed to several factors, including broad-spectrum antimicrobial exposures, poor hand hygiene compliance and horizontal infection control measures, lack of environmental hygiene and use of devices [11–15]. The role of active screening and contact precautions to contain the transmission of this pathogen is still widely debated, as the costs of this operation may outweigh any benefits [11]. A meta-analysis by Prematunge et al. [16] reported increased mortality associated with VRE infections in 2016, but these findings have not been confirmed in any subsequent studies [17, 18]. Confounding factors, such as disease severity and population selection, may explain heterogeneity of results.

VRE infections occur more often in immunocompromised and long-term hospitalized patients [19]. Hematological patients are at higher risk for VRE colonization, as they experience long periods of profound neutropenia [20–22]. Furthermore, some patients undergo induction chemotherapy cycles aimed at hematopoietic stem-cell transplantation (HSCT), experiencing deep immune suppression [23, 24]. As colonization greatly increases the risk for subsequent VRE bloodstream infection (BSI), many studies have been conducted in this specific population [21, 23, 25–30]. However, most studies enrolled patients with particularly severe underlying disease and did not include a control population [21, 23, 27, 31]. Moreover, most studies report VRE infection risk factors only and not predictors of acquired nosocomial VRE colonization [27, 29, 30, 32].

Our study aims to identify risk factors for acquired VRE rectal colonization in hematological patients and to evaluate the clinical impact of VRE colonization on subsequent infection, and 30- and 90-day overall mortality compared to a matched control group.

## Methods

The study was conducted from January 2017 to December 2020 at the Department of Hematology of the University Hospital of Modena, a tertiary hospital with 1,200 beds. During the study period, universal active surveillance was implemented with a rectal swab performed on hospital admission and repeated weekly throughout hospitalization. Contact precautions for all VRE infected / asymptomatic carriers included: (i) single room/cohorting of VRE carriers/functional isolation; (ii) alert code outside the rooms and on the beds, (iii) use of disposable gowns and gloves for all staff; and (iv) disposable or patient-specific intensive care patient dedicated equipment.

Our study was designed as a matched case-control study with case control inclusion at 1:1 ratio. Study criteria specified the inclusion of case patients with nosocomial isolation of a Vancomycin-resistant Enterococcus faecium strain from rectal swab screening (isolate ≥ 48 h from admission) previously negative to a rectal swab at hospital admission and no isolation of VRE from any biological specimen in the preceding 6 months. Detection of VanA and VanB expression was performed by phenotypic methods. Our center’s medical charts of hematological patients admitted to hospital was accessed for matched control selection. Control group selection specified rectal swab negativity at admission, no isolation of VRE from any biological specimen (in the preceding and subsequent 6 months periods), and swab execution dates within the study period. Matching was performed manually for each selected case based on common VRE colonization risk factors, including age, sex, ethnicity, hematological malignancy and stage of hematological illness and hospital stay [20].

Data collected included patient age, sex, hematological disease type and stage, solid organ/bone marrow transplant, comorbidities, specific covariates for the Charlson comorbidity index (CCI) assessment [33], current and previous hospitalization data (dates and time intervals from admission to positive screening, etc.). Altered bowel habits, reduced mobility, cachexia or weight loss were included as intrinsic risk factors for VRE colonization. Use of permanent devices, including indwelling urinary, central venous or peripheral catheters, stents, ostomies and pacemakers, were assessed as extrinsic risk factors for VRE colonization. Presence of altered bowel habits was considered as proxy of intestinal dysbiosis [34, 35]. This study also evaluated previous or current invasive medical procedures and treatments, immunosuppressive therapies (chemotherapy or minimum dose of 0.3 mg/[kg⋅day] equivalent of prednisone for > 3 weeks), administration of proton pump inhibitors and antibiotic exposure before VRE colonization either before (≤ 30 days before admission) or during admission. Since patients with *Clostridiodies difficile* infection (CDI) are at a greater risk of VRE colonization [32, 36, 37], CDI during hospitalization was also collected. A dedicated database with predefined values for data collection from hospital medical charts was created.

This study was approved by the Institutional Ethics Committee (AOU 198/2020/OSS*/*AOUMO). As all data were analyzed anonymously after a deidentification process, no specific written informed patient consent was required.

### Microbiological methods

After samples collection, all isolates were identified by MALDI-TOF MS using VITEK MS (bioMérieux, Marcy l’Etoile, France) following the manufacturer’s instructions. The antimicrobial susceptibility test was performed by the microdilution method using the ITGNEGF antimicrobial susceptibility test panel (MICRONAUT, Merlin, Germany).

### Statistical analysis

Descriptive statistics included categorical variables as proportion (N, percentage [%]), continuous variables (mean ± standard deviation or median and interquartile range [IQR]). Subgroup comparisons were assessed by Unpaired Student’s t or Pearson’s chi-squared tests.

A multivariate logistic regression model, using a stepwise selection, entering the main exposures, and then sequentially all possible confounders (clinically relevant and non-correlated) identified prognostic factors for VRE colonization. All variables were included in the multivariate model in one single step, without checking, and then the non-significant variables were sequentially removed, in a backward stepwise manner.

The intercept-only model was fitted and individual score statistics for potential variables evaluated. P < 0.05 was considered statistically significant. Statistical analysis was performed using STATA® version 14 (StataCorp. 2015. Stata Statistical Software: Release 14. College Station, TX: StataCorp LP.).

## Results

This retrospective, single center study, included 83 consecutive cases of VRE colonization and 83 matched controls. The incidence of nosocomial VRE colonization in the Department of Hematology increased from 2.6 to 4.6 per 1,000 patient-days from 2018 to 2022, with a 10% cumulative incidence of VRE colonization. Of the 83 cases of VRE isolates, 72 were VanA and 11 VanB. The combined study cohort was predominately male 55.4% (n = 92) with an overall median age of 23.6 years (interquartile range 21.3–25.7), see Table 1. The most prevalent hematological diseases were acute myeloid leukaemia (59%), followed by diffuse large B cell lymphoma (10.9%), non-Hodgkin lymphoma (4.8%), Hodgkin’s lymphoma (2.4%), multiple myeloma (7.2%), acute lymphocytic leukaemia (7.2%) and others (8.5%). Moreover, both current and previous hospitalization (≤ 6 months) were significantly associated with VRE colonization.

**Table 1:** Baseline characteristics of patients colonized with VRE matched with patients without VRE colonization (control group)

		Total	Cases	Controls	p-value	OR 95% CI	p-value
		n = 166 (100%)	n = 83 (50%)	n = 83 (50%)			
**Demographic variables**, ***n (%)***							
Gender	Female	74 (44.6)	37 (44.6)	37 (44.6)	1.000	ref.	
	Male	92 (55.4)	46 (55.4)	46 (55.4)		0.31 (0.54–1.84)	1.000
Age, *median, IQR*		23.6. 21.3–25.7	23.6. 21.8–26.0	23.7. 21.1–25.6	0.743	0.99 (0.97–1.01)	0.742
BMI, *mean* ± *SD*		24.4 ± 4.7	23.4 ± 4.5	24.5 ± 4.9	0.875	0.99 (0-93-1.06)	0.874
Leukemia		109 (65.7)	54 (65.1)	55 (66.3)	0.87	0.97 (0.70–1.34)	0.87
Lymphoma		44 (26.5)	24 (28.9)	20 (24.1)	0.482	1.13 (0.80–1.59)	0.482
Hematological disease stage	≤ III	18 (10.8)	10 (12)	8 (9.6)	0.729	ref.	
	> III	28 (16.9)	17 (20.5)	11 (13.3)		1.23 (0.37–4.10)	0.729
Charlson Comorbity Index, *mean ± SD (range)*		5.3 ± 2.3	5.2 ± 2.3	5.4 ± 2.4	0.674	0.97 (0.85–1.10)	0.673
		(1–13)	(1–12)	(1–13)			
**Admission characteristics and provenance**:							
Prior hospitalization (≤ 6 months)		72 (43.4)	49 (59.0)	23 (27.7)	< 0.001	3.75 (1.96–7.20)	**< 0.001**
Current hospitalization *days*, mean ± SD (range)		36.0 ± 28.1	40.7 ± 31.9	31.2 ± 22.7	0.028	1.01 (1.00-1.02)	**0.036**
		(1-259)	(1-115)				
Current hospitalization (prior to colonization), days, mean ± SD (range)		21.8 ± 27.5	21.8 ± 27.5	-	-	-	-
		(1-245)	(1-245)				

OR: odds ratio, CI: confidence interval, IQR: interquartile range, BMI: body mass index, SD: standard deviation, HIV: Human Immunodeficiency Virus

Significant differences in both intrinsic and extrinsic risk factors for VRE colonization were observed. Significant intrinsic risk factors included febrile neutropenia, bone marrow transplant, peptic ulcer disease, Chronic Obstructive Pulmonary Disease, cachexia, reduced mobility and altered bowl habits. Significant extrinsic risk factors included central venous catheter and presence of surgical wounds / bedsores, see Table 2.

**Table 2:** Univariate analysis of Intrinsic and extrinsic risk factors associated with VRE nosocomial rectal colonization

Risk factors, *n (%)*			Total (n = 166)			Cases, n = 83 (50%)	Controls, n = 83 (50%)	p-value	OR 95% CI	p-value
**Intrinsic**										
Hematological status:										
Bone marrow transplant			23 (13.9)			16 (19.3)	7 (8.4)	0.043	2.59 (1.00-6.68)	**0.049**
Febrile neutropenia			109 (65.7)			62 (74.7)	47 (56.6)	0.014	2.26 (1.17–4.36)	**0.015**
Comorbidities:										
Dementia			6 (3.6)			4 (4.8)	2 (2.4)	0.406	2.05 (0.36–11.51)	0.415
Hypertension			76(45.8)			42 (50.6)	34 (41)	0.213	1.47 (0.79–2.72)	0.213
Congestive Heart Failure			34 (20.5)			14 (16.9)	20 (24.1)	0.249	0.63 (0.29–1.37)	0.251
Chronic Obstructive Pulmonary Disease			11 (6.6)			1 (1.2)	10 (12)	0.005	0.08 (0.01–0.71)	**0.023**
Peptic ulcer disease			16 (9.6)			12 (14.5)	4 (4.8)	0.035	3.33 (1.02–10.82)	**0.045**
Liver disease			40 (24.1)			22 (26.5)	18 (21.7)	0.468	1.37 (0.66–2.84)	0.384
Diabetes Mellitus			25 (15.1)			12 (14.5)	13 (15.7)	0.828	0.91 (0.38–2.13)	0.828
Chronic Kidney Disease			10 (6.0)			4 (4.8)	6 (7.2)	0.514	0.80 (0.42–1.54)	0.517
Peripheral Vascular Disease			64 (40.4)			34 (41)	30 (36)	0.874	1.05 (0.56–1.95)	0.874
Solid Tumor			19 (11.4)			6 (7.2)	13 (15.7)	0.088	0.64 (0.38–1.07)	0.095
Solid transplant			4 (2.4)			2 (2.4)	2 (2.4)	1.000	1.0 (0.13–7.27)	1.000
Other predisposing factors to colonization										
Cachexia or weight loss			26 (15.7)			18 (21.7)	8 (9.6)	0.033	2.59 (1.05–6.36)	**0.037**
Reduced mobility			41 (24.7)			27 (32.5)	14 (16.9)	0.019	2.37 (1.13–4.95)	**0.021**
Altered bowel habits			93 (56.0)			59 (71.1)	34 (41)	< 0.001	3.54 (1.85–6.75)	**< 0.001**
Colonization status:										
MDR pathogens:			37 (22.3)			18 (21.7)	19 (22.9)	0.852		
XDR *P. aeruginosa*			9 (5.4)			4 (4.8)	5 (6)	0.775	0.78 (0.20–3.05)	0.732
*E. coli* ESBL+			6 (3.6)			4 (4.8)	2 (2.4)		1.47 (0.24–9.11)	0.673
CPE			3 (1.8)			1 (1.2)	2 (2.4)		1.97 (0-17-22.23)	0.582
CRAB			1 (0.6)			0 (0.0)	1 (1.2)		1 (empty)	
MRSA			1 (0.6)			0 (0.0)	1 (1.2)		1 (empty)	
**Extrinsic**										
Presence of an invasive device/intervention:										
Pacemaker			3 (1.8)			1 (1.2)	2 (2.4)	0.560	0.49 (0.04–5.55)	0.568
Prosthesis/stent			13 (7.8)			3 (3.6)	10 (12)	0.043	0.27 (0.07–1.03)	0.056
Central venous catheter			138 (83.1)			78 (94)	60 (72.3)	< 0.001	5.97 (2.14–16.65)	**0.001**
Tracheostomy			5 (3.0)			3 (3.6)	2 (2.4)	0.650	1.51 (0.24–9.33)	0.652
Urinary catheter			66 (39.8)			38 (45.8)	28 (33.7)	0.113	1.65 (0.88–3.10)	0.114
Non-abdominal surgery ($\le$6 months)			21 (12.7)			14 (16.9)	7 (8.4)	0.102	2.20 (0.84–5.77)	0.108
Surgical wounds/ pressure ulcers			48 (28.9)			34 (41.0)	14 (16.9)	0.001	3.41 (1.66–7.04)	**0.001**
Abdominal surgery ($\le$6 months)			84 (50.6)			44 (53.0)	40 (48.2)	0.535	1.21 (0.65–2.23)	0.535

OR: odds ratio, CI: confidence interval, BMI: body mass index, HIV: Human Immunodeficiency Virus, MDR: Multi Drug Resistant, XDR: eXtensively Drug Resistant,

ESBL: Extended Spectrum Beta-Lactamase, CPE: Carbapenemase Producing Enterobacterales, CRAB: Carbapenem Resistant Acinetobacter Baumannii, MRSA:

Methicillin Resistant Staphylococcus aureus, CVC: Central Venous Catheter, PICC: Peripherally Inserted Central Catheter

According to treatments administered, there were significant differences among the groups in corticosteroids and antibiotics, both ≤ 6 months or during (regardless of class) hospitalization, see Table 3 and Supplementary Table 1. Specifically, prior administration of vancomycin, ceftriaxone, piperacillin-tazobactam and linezolid, and administration during hospitalization of vancomycin and ceftriaxone were significantly different. All therapieswere confirmed risk factors for VRE colonization at univariable analysis. Of clinical relevance, prior and concomitant (oral/intravenous) administration of vancomycin (but not teicoplanin) was identified.

**Table 3:** Frequency and univariate analysis of previous and concurrent treatments associated with VRE nosocomial rectal colonization

	Frequency				Univariate analysis	
	Total (n = 166)	Cases, n = 83 (50%)	Controls, n = 83 (50%)	p-value	OR 95% CI	p-value
**Prior to hospitalization**, ***n (%)***						
Use of antibiotics ($\le$ 6 month)	84 (50.6)	53 (63.9)	31 (37.0)	**0.001**	2.96 (1.57–5.57)	**0.001**
Vancomycin	16 (19.0)	14 (26.0)	2 (7.0)	**0.025**	5.20 (1.09–24.7)	**0.038**
Teicoplanin	1 (1.0)	1 (2.0)	-	0.442	1 (omitted)	-
Ceftriaxone	12 (14.0)	11 (21.0)	1 (3.0)	**0.027**	7.85 (0.96–64.16)	0.054
Cefepime	2 (2.0)	-	2 (7.0)	0.061	1 (omitted)	-
Levofloxacin	29 (35.0)	20 (38.0)	9 (29.0)	0.418	1.48 (0.57–3.84)	0.419
Piperacillin-Tazobactam	35 (42.0)	27 (51.0)	8 (26.0)	**0.024**	2.98 (1.13–7.86)	**0.027**
Meropenem	19 (23.0)	15 (28.0)	4 (13.0)	0.104	2.66 (0.79–8.91)	0.112
Tigecycline	2 (2.0)	1 (2.0)	1 (3.0)	0.698	0.57 (0.03–9.56)	0.701
Daptomycin	3 (4.0)	3 (6.0)	-	0.177	1 (omitted)	-
Linezolid	11 (13.0)	10 (19.0)	1 (3.0)	**0.04**	6.97 (0.84–57.4)	0.071
**Current hospitalization**, ***n(%)***						
Use of PPI	130 (78.3)	64 (77.1)	66 (80.0)	0.706	0.86 (0.41–1.81)	0.707
Use of corticosteroids	115 (69.3)	65 (78.3)	50 (60.0)	**0.012**	2.38 (1.20–4.71)	**0.013**
Antibiotic prophylaxis	117 (70.5)	62 (74.7)	55 (66.0)	0.234	1.50 (0.76–2.94)	0.235
Antibiotic therapy	148 (89.2)	81 (97.6)	67 (81.0)	**< 0.001**	9.67 (2.14–43.57)	**0.003**
**Vancomycin**, ***n (%)***						
Vancomycin (oral/intravenous)	68 (41.0)	43 (51.8)	25 (30.0)	**0.004**	2.49 (1.31–4.71)	**0.005**
Vancomycin (oral only)	8 (4.8)	4 (4.8)	4 (4.8)	0.459	0.57 (o.13-2.52)	0.463
Vancomycin duration, *days, mean*$\pm$*SD (range)*	2.7 ± 4.8 (1–20)	2.9 ± 4.7 (1–18)	2.5 ± 4.9 (1–20)	0.574	1.01 (0.95–1.08)	0.572
**Third generation cephalosporins**	36 (24,3)	25 (30,9)	11 (16,4)	**0.041**	2.27 (1.02–5.05)	**0.044**

OR: odds ratio, CI: confidence interval, PPI: proton pump inhibitors

As shown in Table 4, VRE colonization resulted as a significant risk factor for VRE infection, which occurred in 11 cases vs. zero events among controls (p = 0.001), with a high prevalence of bacteremia (8 out of 11). The median time from colonization to development of infection was 30 days. VRE colonization and eventual infection did not influence overall 30 or 90 day mortality rates. Furthermore, almost all cases of CDI were observed in VRE colonized patients (7/8 patients), p = 0.030. Further analysis revealed that CDI was observed in patients prior to VRE colonization in 67% of the cases with a median time to VRE colonization of 66 days (data not shown). Multivariable regression analysis identified any use of vancomycin and altered bowl habits as independent risk factors for nosocomial rectal VRE colonization. Increased risks were > 3 times in patients with any use of vancomycin (OR = 3.5; 95%CI: 1.15–10.87; p = 0.027) or altered bowel habits (OR = 3.1; 95%CI: 1.07–8.94; p = 0.036), > 7 times in patients treated with third generation cephalosporins (OR = 7.7; 95%CI: 0.87–67.99; p = 0.067) and > 2 times in patients with bone marrow transplant (OR: 2.3; 95%CI: 0.65–8.08; p = 0.200), see Table 5.

**Table 4:** Outcomes of patients colonized with VRE compared with those of controls

	Total (n = 166)		Cases. n = 83 (50%)		Controls. n = 83 (50%)		p-value		
**VRE infection**	11 (6.6)		11 (13.3)		0 (0.0)		**0.001**		
BSI	8 (4.8)		8 (9.6)		0 (0.0)				
UTI	2 (1.2)		2 (2.4)		0 (0.0)				
IAI	1 (0.6)		1 (1.2)		0 (0.0)				
**No VRE infection**	155 (93.4)		72 (86.7)		83 (100.0)				
**Time from VRE colonization to VRE infection** *days, mean* $\pm$ *SD (range)*			30.2, 26.8(0–72)						
**CDI**	8 (4.8)		7 (8.4)		1 (1.2)		**0.030**		
**30-day mortality**, ***n/N (%)***									
Overall *		10/166 (6.0)		4 (4.8)		6 (7.2)		0.514	
VRE infection		0/11 (0.0)		0/11 (0.0)		0/0 (0.0)		-	
No VRE infection		10/155 (6.4)		4/72 (5.6)		6/83 (7.2)		0.672	
**90-day mortality**, ***n/N (%)***		20 /166 (12.0)		8 (9.6)		12 (14.5)		0.340	

VRE: vancomycin resistant Enterococcus, BSI: bloodstream infection, UTI: urinary tract infection, IAI: intra-abdominal infection, CDI: *Clostridioides difficile* infection

*comparison between 30 day mortality of cases with or without VRE infection, p_0.448

**Table 5:** Multivariate analysis of risk factors for VRE nosocomial rectal colonization in hematological patients

	OR	95% CI	p-value	
Any use of vancomycin	3.5	(1.15–10.87)	**0.027**	
Use of third generation cephalosporins	7.7	(0.87–67.99)	0.067	
Bone marrow transplant	2.3	(0.65–8.08)	0.200	
Altered bowel habits	3.1	(1.07–8.94)	**0.036**	
Hospitalization in the previous 6 months	2.3	(0.93–5.43)	0.170	

OR: odds ratio, CI: confidence interval

## Discussion

Our study reports that any use of vancomycin and altered bowel habits are the main predisposing factors for nosocomial VRE colonization among hematological patients. Moreover, VRE infection is more likely in patients with rectal nosocomial acquired VRE colonization compared to those without.

Our study has highlighted many intrinsic and extrinsic factors that are associated with nosocomial VRE colonization. Awareness of these factors may assist in improving target screening strategies for early identification of patients at risk [20], limit nosocomial VRE spread, and consequent invasive VRE infections. As routine screening of hematological patients for VRE colonization does not seem cost-effective, screening of high-risk individuals seems paramount [38].

Previous unmatched studies have reported that bone marrow transplant and febrile neutropenia are risk factors for VRE colonization in hematological patients [23, 32, 39, 40]. In our matched case-control study, bone marrow transplant and febrile neutropenia were both proven to be independent risk factors, regardless of the stage of hematological disease. Moreover, some extrinsic elements, such as appropriate devices use (temporary or permanent), careful handling of dressings and bedsores, may assist in the prevention of central line catheter and biliary stents infections and their early removal could prevent VRE hospital transmission [41, 42]. This finding supports the assumption of VRE environmental contamination and its great biofilm forming ability [43]. In addition, bedsores, altered bowel habits, cachexia and reduced mobility were identified as risk factors for VRE colonization, suggesting that more attention must be given to bedridden hematological patients. Furthermore, altered bowel habits was the only intrinsic risk factor associated with VRE colonization in our multivariate analysis.

As already described in literature [7, 16, 21, 22, 32, 39], altered bowel habits may reflect gastrointestinal disruption derived from antibiotic therapy. However, many other causes may also contribute in a hematological nosocomial setting (i.e. chemotherapies, neutropenia and corticosteroids use). The importance of gastrointestinal disturbance as a leading cause of VRE acquisition has already been highlighted by Webb et al. who developed a predictive score for VRE BSI in patients with hematological malignancy [32].

It is still debated whether vancomycin itself, rather than the duration or the route of administration, may increase the risk of VRE colonization [12, 20]. Our data show an increased risk regardless of the route of administration or therapy duration. This finding confirms the independent role of vancomycin in VRE acquisition, as previously suggested by Nerandzic et al. [44]. Recently, Guarana et al. demonstrated that septic shock or early death was not associated with Gram-positive bacteremia. Together with our findings, current guideline recommendations for the empirical use of vancomycin as first line therapy for neutropenic fever, may be challenged [45, 46].

In our hematological hospital setting, also previous usages of certain antibiotics are associated with VRE colonization. As prior studies have underlined, previous use of cephalosporins and piperacillin/tazobactam are associated with an increased risk of VRE colonization [47]. These data emphasize the need to implement antimicrobial stewardship interventions, targeting broad spectrum antibiotics to complement infection control procedures against VRE [48]. Finally, among cases, we found a significant increased use of linezolid in the previous 6 months, while, unexpectedly, VRE colonization does not seem to influence the increased use of this drug during the current hospitalization compared to controls [49]. Linezolid should be reserved for patients at high risk of VRE infection or those with nosocomial pneumonia, as it is the best available antibiotic option for VRE [31, 46].

Our study confirms the ever-growing evidence of microbial interaction between VRE and CDI [50]. However, it is still a matter of debate whether previous vancomycin therapy for CDI is an independent risk factor for VRE colonization or, alternatively, whether VRE gut colonization enhances fitness and pathogenesis of *C. difficile*. Our results seem to underline the supportive role of pathogenic microbiota, as a common immunopathogenic mechanism.

In hematological patients, exposure to chemotherapy, underlying neutropenia and use of broad-spectrum antibiotics are risk factors for mucositis and intestinal microbiota alterations. This increased dysregulation led to CDI, subsequent VRE colonization and translocation into the bloodstream resulting in bacteremia. Indeed, *C. difficile* and VRE have both been shown to be agents responsible for Graft Versus Host Disease (GvHD) and, more generally, for bone marrow transplant failure [25, 27, 28, 32, 46]. Our findings appear even more relevant considering that new treatment strategies for altered bowel habits and CDI are being developed, such as oral microbiome therapy [51].

The clinical impact in terms of mortality between VRE and vancomycin-sensitive Enterococcus is still debated in literature, and, in particular, whether a higher mortality is attributable to the pathogen itself or progression of the hematological disease [16, 52]. Interestingly, in contrast with other studies conducted in similar populations [13, 14, 17, 18, 29, 30, 53–55], our data suggests that there is no difference in mortality between colonized patients and in the subgroup population who developed a VRE infection (often BSI). However, these previous studies of VRE often have included both *E. faecium* and *E. faecalis*, coming from a complex mix of patient populations among different countries. When adjusted for species, vancomycin-resistance seems not to further increase the risk of clinical failure. Indeed, our data are in line with other recently published studies conducted in other settings, such as liver transplant or abdominal surgery patients, where vancomycin resistance does not seem to influence outcome [17, 18]. Nevertheless, given the relatively low number of infections, these data should be interpreted with caution.

Our study has several limitations. The single-center retrospective study design limits the generalizability of our results. Furthermore, retrospective data collection did not allow any investigations into the best approaches for VRE prevention. However, as our center implemented a universal screening policy, data enabled the calculation of nosocomial prevalence rates. Further, there is an innate selection bias associated with a case–control methodological approach. However, we tried to limit this bias by selected a matching criterion based on commonly accepted risk factors previously identified in literature.

## Conclusion

Risk factors for VRE nosocomial acquisition among hematological patients identified in this study include any use of vancomycin and altered bowel habits. VRE nosocomial colonization prevention in a hematological setting urgently requires an antimicrobial stewardship strategy, focused on reducing inadequate Gram-positive coverage. A gastrointestinal barrier damage may be more pronounced in hematological patients, which may account for the different pathogenicity of VRE compared to other clinical settings. However, VRE colonization and VRE infection do not seem to be associated with increased 30- and 90-day mortality. Finally, the strong correlation between CDI and VRE deserves further investigation, also in other healthcare settings, to target new approaches of prevention and treatment.

### Electronic supplementary material

Below is the link to the electronic supplementary material.

Supplementary Material 1

## Data Availability

The datasets used and/or analyzed during the current study are available from the corresponding author on reasonable request.

## References

[1] Andrewes FW, Horder TJ, A STUDY OF, THE STREPTOCOCCI PATHOGENIC FOR MAN (1906). The Lancet.

[2] Crank C, O’Driscoll T. Vancomycin-resistant enterococcal Infections: epidemiology, clinical manifestations, and optimal management. Infect Drug Resist. 2015;217. 10.2147/IDR.S54125 PMC4521680 26244026

[3] Pfaller MA, Cormican M, Flamm RK, Mendes RE, Jones RN (2019). Temporal and Geographic Variation in Antimicrobial susceptibility and resistance patterns of Enterococci: results from the SENTRY Antimicrobial Surveillance Program, 1997–2016. Open Forum Infect Dis.

[4] Faron ML, Ledeboer NA, Buchan BW (2016). Resistance mechanisms, Epidemiology, and approaches to Screening for Vancomycin-resistant Enterococcus in the Health Care setting. J Clin Microbiol.

[5] Courvalin P (2006). Vancomycin Resistance in Gram-positive Cocci. Clin Infect Dis.

[6] Kankalil George S, Suseela MR, El Safi S, Ali Elnagi E, Al-Naam YA, Adlan Mohammed Adam A (2021). Molecular determination of van genes among clinical isolates of enterococci at a hospital setting. Saudi J Biol Sci.

[7] Harbarth S, Cosgrove S, Carmeli Y (2002). Effects of Antibiotics on Nosocomial Epidemiology of Vancomycin-Resistant Enterococci. Antimicrob Agents Chemother.

[8] World Health Organization., https://www.who.int/news/item/27-02-2017-who-publishes-list-of-bacteria-for-which-new-antibiotics-are-urgently-needed. Accessed 5 April 2023.

[9] WHO Regional Office for Europe/European Centre for Disease Prevention and Control (2022). Antimicrobial resistance surveillance in Europe 2022–2020 data.

[10] Zhou X, Willems RJL, Friedrich AW, Rossen JWA, Bathoorn E (2020). Enterococcus faecium: from microbiological insights to practical recommendations for Infection control and diagnostics. Antimicrob Resist Infect Control.

[11] Morgan DJ, Pineles L, Shardell M, Graham MM, Mohammadi S, Forrest GN (2013). The Effect of Contact precautions on Healthcare Worker Activity in Acute Care hospitals. Infect Control Hosp Epidemiol.

[12] Sakka V, Tsiodras S, Galani L, Antoniadou A, Souli M, Galani I (2008). Risk-factors and predictors of mortality in patients colonised with Vancomycin-resistant enterococci. Clin Microbiol Infect.

[13] López-Luis BA, Sifuentes-Osornio J, Lambraño-Castillo D, Ortiz-Brizuela E, Ramírez-Fontes A, Tovar-Calderón YE (2021). Risk factors and outcomes associated with Vancomycin-resistant Enterococcus faecium and ampicillin-resistant Enterococcus faecalis bacteraemia: a 10-year study in a tertiary-care centre in Mexico City. J Glob Antimicrob Resist.

[14] Billington EO, Phang SH, Gregson DB, Pitout JDD, Ross T, Church DL (2014). Incidence, risk factors, and outcomes for Enterococcus spp. Blood Stream Infections: a Population-based study. Int J Infect Dis.

[15] Kampmeier S, Kossow A, Clausen LM, Knaack D, Ertmer C, Gottschalk A (2018). Hospital acquired Vancomycin resistant enterococci in surgical intensive care patients – a prospective longitudinal study. Antimicrob Resist Infect Control.

[16] Prematunge C, MacDougall C, Johnstone J, Adomako K, Lam F, Robertson J (2016). VRE and VSE Bacteremia Outcomes in the era of effective VRE therapy: a systematic review and Meta-analysis. Infect Control Hosp Epidemiol.

[17] Dubler S, Lenz M, Zimmermann S, Richter DC, Weiss KH, Mehrabi A (2020). Does Vancomycin resistance increase mortality in Enterococcus faecium bacteraemia after orthotopic liver transplantation? A retrospective study. Antimicrob Resist Infect Control.

[18] Kramer TS, Remschmidt C, Werner S, Behnke M, Schwab F, Werner G (2018). The importance of adjusting for enterococcus species when assessing the burden of Vancomycin resistance: a cohort study including over 1000 cases of enterococcal bloodstream Infections. Antimicrob Resist Infect Control.

[19] Hayden MK (2000). Insights into the Epidemiology and Control of Infection with Vancomycin-Resistant Enterococci. Clin Infect Dis.

[20] Alevizakos M, Gaitanidis A, Nasioudis D, Tori K, Flokas ME, Mylonakis E. Colonization with vancomycin-resistant enterococci and risk for bloodstream Infection among patients with malignancy: a systematic review and Meta-analysis. Open Forum Infect Dis. 2017;4(1). 10.1093/ofid/ofw246 PMC5414102 28480243

[21] Ford CD, Lopansri BK, Haydoura S, Snow G, Dascomb KK, Asch J (2015). Frequency, risk factors, and outcomes of vancomycin-resistant *Enterococcus* colonization and Infection in patients with newly diagnosed Acute Leukemia: different patterns in patients with Acute Myelogenous and Acute Lymphoblastic Leukemia. Infect Control Hosp Epidemiol.

[22] Worth LJ, Thursky KA, Seymour JF, Slavin MA (2007). Vancomycin-resistant Enterococcus faecium Infection in patients with hematologic malignancy: patients with acute Myeloid Leukemia are at high-risk. Eur J Haematol.

[23] Weinstock DM, Conlon M, Iovino C, Aubrey T, Gudiol C, Riedel E (2007). Colonization, Bloodstream Infection, and Mortality caused by vancomycin-resistant Enterococcus early after allogeneic hematopoietic stem cell transplant. Biol Blood Marrow Transplant.

[24] Ziakas PD, Pliakos EE, Zervou FN, Knoll BM, Rice LB, Mylonakis E (2014). MRSA and VRE colonization in solid organ transplantation: a Meta-analysis of published studies. Am J Transplant.

[25] Ford CD, Gazdik MA, Lopansri BK, Webb B, Mitchell B, Coombs J (2017). Vancomycin-resistant Enterococcus colonization and bacteremia and hematopoietic stem cell transplantation outcomes. Biol Blood Marrow Transplant.

[26] Weber DJ, Anderson D, Rutala WA (2013). The role of the surface environment in healthcare-associated Infections. Curr Opin Infect Dis.

[27] Kang Y, Vicente M, Parsad S, Brielmeier B, Pisano J, Landon E et al. Evaluation of risk factors for Vancomycin-resistant *Enterococcus* bacteremia among previously colonized hematopoietic stem cell transplant patients. Transpl Infect Disease. 2013;n/a-n/a 10.1111/tid.12120 23911080

[28] Rosko AE, Corriveau M, Suwantarat N, Arfons L, Treasure M, Parker P (2014). Vancomycin-resistant enterococci Infection: not just for the transplanted. Leuk Lymphoma.

[29] Kirkizlar TA, Akalin H, Kirkizlar O, Ozkalemkas F, Ozkocaman V, Kazak E (2020). Vancomycin-resistant enterococci Infection and predisposing factors for Infection and mortality in patients with acute Leukaemia and febrile neutropenia. Leuk Res.

[30] Papanicolaou GA, Ustun C, Young JAH, Chen M, Kim S, Woo Ahn K (2019). Bloodstream Infection due to Vancomycin-resistant Enterococcus is Associated with increased mortality after hematopoietic cell transplantation for Acute Leukemia and Myelodysplastic Syndrome: a Multicenter, Retrospective Cohort Study. Clin Infect Dis.

[31] Kamboj M, Cohen N, Huang YT, Kerpelev M, Jakubowski A, Sepkowitz KA (2019). Impact of empiric treatment for vancomycin-resistant Enterococcus in colonized patients early after allogeneic hematopoietic stem cell transplantation. Biol Blood Marrow Transplant.

[32] Webb BJ, Healy R, Majers J, Burr Z, Gazdik M, Lopansri B (2017). Prediction of bloodstream Infection due to vancomycin-resistant Enterococcus in patients undergoing Leukemia induction or hematopoietic stem-cell transplantation. Clin Infect Dis.

[33] Charlson M, Szatrowski TP, Peterson J, Gold J (1994). Validation of a combined comorbidity index. J Clin Epidemiol.

[34] Singh R, Zogg H, Wei L, Bartlett A, Ghoshal UC, Rajender S (2021). Gut Microbial Dysbiosis in the Pathogenesis of Gastrointestinal Dysmotility and Metabolic disorders. J Neurogastroenterol Motil.

[35] D’Angelo G. Microbiota and Hematological Diseases. Int J Hematol Oncol Stem Cell Res. 2022. 10.18502/ijhoscr.v16i3.10139 PMC9831866 36694706

[36] Stevens VW, Khader K, Echevarria K, Nelson RE, Zhang Y, Jones M (2020). Use of oral vancomycin for Clostridioides difficile Infection and the risk of vancomycin-resistant Enterococci. Clin Infect Dis.

[37] Ling R, Achonu C, Li Y, Katz KC, Garber G, Johnstone J (2020). Investigating the relationship between Vancomycin-resistant Enterococcus control practices and the incidence of health care–associated Clostridioides difficile Infections in Ontario. Am J Infect Control.

[38] Gedik H, Şimşek F, Yıldırmak T, Kantürk A, Aydın D, Demirel N (2015). Which multidrug-resitant Bacteria are emerging in patients with hematological malignancies? One-year report. Indian J Hematol Blood Transfus.

[39] DiazGranados CA, Jernigan JA (2005). Impact of Vancomycin Resistance on Mortality among patients with Neutropenia and Enterococcal Bloodstream Infection. J Infect Dis.

[40] White L, Ybarra M (2017). Neutropenic Fever. Hematol Oncol Clin North Am.

[41] Böll B, Schalk E, Buchheidt D, Hasenkamp J, Kiehl M, Kiderlen TR (2021). Central venous catheter–related Infections in hematology and oncology: 2020 updated guidelines on diagnosis, management, and prevention by the Infectious Diseases Working Party (AGIHO) of the German Society of Hematology and Medical Oncology (DGHO). Ann Hematol.

[42] Viola GM, Szvalb AD, Malek AE, Chaftari A, Hachem R, Raad II (2023). Prevention of device-related Infections in patients with cancer: current practice and future horizons. CA Cancer J Clin.

[43] Barber KE, Shammout Z, Smith JR, Kebriaei R, Morrisette T, Rybak MJ (2021). Biofilm Time-kill curves to assess the bactericidal activity of Daptomycin combinations against Biofilm-Producing Vancomycin-resistant Enterococcus faecium and faecalis. Antibiotics.

[44] Nerandzic MM, Mullane K, Miller MA, Babakhani F, Donskey CJ (2012). Reduced Acquisition and Overgrowth of Vancomycin-Resistant Enterococci and Candida Species in patients treated with Fidaxomicin Versus Vancomycin for Clostridium difficile Infection. Clin Infect Dis.

[45] Guarana M, Nucci M, Nouér SA. Shock and early death in hematologic patients with Febrile Neutropenia. Antimicrob Agents Chemother. 2019;63(11). 10.1128/AAC.01250-19 PMC6811434 31405857

[46] Freifeld AG, Bow EJ, Sepkowitz KA, Boeckh MJ, Ito JI, Mullen CA (2011). Clinical practice Guideline for the Use of Antimicrobial agents in Neutropenic patients with Cancer: 2010 update by the Infectious Diseases Society of America. Clin Infect Dis.

[47] Xie O, Slavin MA, Teh BW, Bajel A, Douglas AP, Worth LJ (2020). Epidemiology, treatment and outcomes of bloodstream Infection due to Vancomycin-resistant enterococci in cancer patients in a vanB endemic setting. BMC Infect Dis.

[48] Gouliouris T, Warne B, Cartwright EJP, Bedford L, Weerasuriya CK, Raven KE (2018). Duration of exposure to multiple antibiotics is associated with increased risk of VRE bacteraemia: a nested case-control study. J Antimicrob Chemother.

[49] Short E, Esterly J, Postelnick M, Ong J, McLaughlin M (2014). Disposition of linezolid or daptomycin in Enterococcal bloodstream Infections according to Vancomycin resistant Enterococcus colonization. Antimicrob Resist Infect Control.

[50] Smith AB, Jenior ML, Keenan O, Hart JL, Specker J, Abbas A (2022). Enterococci enhance Clostridioides difficile pathogenesis. Nature.

[51] Feuerstadt P, Louie TJ, Lashner B, Wang EEL, Diao L, Bryant JA (2022). SER-109, an oral Microbiome Therapy for recurrent *Clostridioides difficile* Infection. N Engl J Med.

[52] Choi PYI, Straube B, Cook C, Van Der Weyden C, Ramanathan S, Taylor P (2011). The mortality of vancomycin-resistant Enterococci Bloodstream Infections (VRE BSI). Blood.

[53] Hemapanpairoa J, Changpradub D, Thunyaharn S, Santimaleeworagun W (2021). Does Vancomycin Resistance increase mortality? Clinical outcomes and predictive factors for mortality in patients with Enterococcus faecium Infections. Antibiotics.

[54] Rao C, Dhawan B, Vishnubhatla S, Kapil A, Das B, Sood S (2020). Emergence of high-risk multidrug-resistant Enterococcus faecalis CC2 (ST181) and CC87 (ST28) causing healthcare-associated Infections in India. Infect Genet Evol.

[55] Alatorre-Fernández P, Mayoral-Terán C, Velázquez-Acosta C, Franco- Rodríguez C, Flores-Moreno K, Cevallos MÁ (2017). A polyclonal outbreak of bloodstream Infections by Enterococcus faecium in patients with hematologic malignancies. Am J Infect Control.

