# Supplementary material for: Vancomycin resistant enterococcus risk factors for hospital colonization in hematological patients: a matched case-control study
Source: Antimicrob Resist Infect Control. 2023 Nov 13;12:126. doi: 10.1186/s13756-023-01332-x (PMC10644555; doi:10.1186/s13756-023-01332-x)
Supplement: Supplementary file 1 — Supplementary Material 1 [file 13756_2023_1332_MOESM1_ESM.docx]

*Appendix A. Supplementary data*

Table 1. Antibiotic exposure as treatment during hospitalization comparing cases with controls.

|  | **Total (n=148)** | **Cases, n=81 (54.7%)** | **Controls, n=67 (43.3%)** | **p-value** |
| --- | --- | --- | --- | --- |
| **Antibiotic exposure as treatment during hospitalization** | | | |  |
| **Total duration of antibiotic treatment during hospitalization** | 28.9 ±27.5(0-233) | 32.0 ±33.9(3-233) | 25.1 ±16.3(0-72) | 0.130 |
| **Number of antibiotics used in the hospitalization, mean ± SD (range)** | 3.6 ±1.9(1-7) | 3.7 ±1.7(1-7) | 3.5 ±2.1(1-7) | 0.493 |
| **1 VANCOMYCIN** | 68 (41.0) | 43 (51.8) | 25 (30.0) | **0.004** |
| **2 TEICOPLANIN** | 0 (0) | 0 (0) | 0 (0) | - |
| **3 CEFTRIAXONE** | 36 (24,3) | 25 (30,9) | 11 (16,4) | **0.041** |
| **4 CEFEPIME** | 1 (0,7) | 1 (1,2) | 0 (0) | 0.361 |
| **5 CEFTAZIDIME** | 2 (1,4) | 1 (1,2) | 1 (1,5) | 0.892 |
| **6 LEVOFLOXACIN** | 96 (64,9) | 50 (61,7) | 46 (68,7) | 0.379 |
| **7 PIPERACILLIN-TAZOBACTAM** | 90 (60,8) | 50 (61,7) | 40 (59,7) | 0.801 |
| **8 MEROPENEM** | 58 (39,2) | 29 (35,8) | 29 (43,3) | 0.353 |
| **9 TIGECYCLINE** | 5 (3,4) | 3 (3,7) | 2 (3) | 0.810 |
| **10 DAPTOMYCIN** | 1 (0,7) | 1 (1,2) | 0 (0) | 0.361 |
| **11 LINEZOLID** | 26 (17,6) | 12 (14,8) | 14 (20,9) | 0.333 |
| **12 AMOXICILLIN-CLAVULANATE** | 2 (1,4) | 1 (1,2) | 1 (1,5) | 0.982 |
| **13 TRIMETHOPRIM-SULFAMETHOXAZOLE** | 31 (20,9) | 16 (19,8) | 15 (22,4) | 0.695 |
| **14 CIPROFLOXACIN** | 2 (1,4) | 2 (2,5) | 0 (0) | 0.195 |
| **15 AZITHROMICIN** | 9 (6,1) | 5 (6,2) | 4 (6) | 0.959 |
| **16 AMIKACIN** | 20 (13,5) | 10 (12,3) | 10 (14,9) | 0.648 |
| **17 CLINDAMICYCIN** | 1 (0,7) | 0 (0) | 1 (1,5) | 0.270 |
| **18 METRONIDAZOLE** | 0 (0) | 0 (0) | 0 (0) | 0.433 |
